# Coverage with Selected Vaccines and Exemption Rates Among Children in Kindergarten — United States, 2023–24 School Year

**DOI:** 10.15585/mmwr.mm7341a3

**Published:** 2024-10-17

**Authors:** Ranee Seither, Oyindamola Bidemi Yusuf, Devon Dramann, Kayla Calhoun, Agnes Mugerwa-Kasujja, Cynthia L. Knighton, Jennifer L. Kriss, Rebecca Miller, Georgina Peacock

**Affiliations:** ^1^Immunization Services Division, National Center for Immunization and Respiratory Diseases, CDC; ^2^Certified Technical Experts, Inc., Montgomery, Alabama; ^3^Association of Schools and Programs of Public Health, Washington, DC.

**Summary**

**What is already known about this topic?**

From the 2019–20 to the 2022–23 school year, national kindergarten coverage with state-required vaccinations declined from 95% to approximately 93%.

**What is added by this report?**

During the 2023–24 school year, coverage declined to <93% for all reported vaccines (range = 92.3% [diphtheria, tetanus, and acellular pertussis vaccine] to 92.7% [measles, mumps, and rubella vaccine]). The exemption rate increased to 3.3% from 3.0% the year before and increased in 41 jurisdictions, exceeding 5% in 14.

**What are the implications for public health practice?**

Decreasing vaccination coverage and increasing exemptions increase the risk for vaccine-preventable disease outbreaks. Efforts by health departments, schools, and providers are needed to ensure that students begin school fully vaccinated.

## Abstract

In the United States, states and local jurisdictions set vaccination requirements for school attendance, conditions and procedures for exemptions from these requirements, grace periods for submitting documentation, and provisional enrollment for students who need more time to be vaccinated. States annually report data to CDC on the number of children in kindergarten who meet, are exempt from, or are in the process of meeting requirements. Data reported by 49 states and the District of Columbia (DC) for the 2023–24 school year were used for national- and state-level estimates of the following measures: complete vaccination with required doses of measles, mumps, and rubella vaccine (MMR), diphtheria, tetanus, and acellular pertussis vaccine (DTaP), poliovirus vaccine (polio), and varicella vaccine (VAR); exemptions from vaccination; and school attendance while meeting requirements. The 2023–24 kindergarten class became age-eligible to complete most state-required vaccinations during the COVID-19 pandemic, after schools had returned to routine in-person learning. Compared with approximated national coverage levels across all reported vaccines for the 2019–20 (95%) and 2022–23 (93%) school years, coverage dropped below 93% for the 2023–24 school year, ranging from 92.3% for DTaP to 92.7% for MMR. Exemptions increased to 3.3%, compared with those during the 2022–23 (3.0%) and 2021–22 school years (2.6%). Coverage with MMR, DTaP, polio, and VAR decreased in 35, 32, 33, and 36 jurisdictions, respectively, compared with the 2022–23 school year. Exemptions increased in 41 jurisdictions, with 14 reporting that >5% of kindergartners had an exemption from one or more vaccine. Efforts by health departments, schools, and providers are needed to ensure that students begin school fully vaccinated.

## Introduction

School vaccination requirements set by state and local jurisdictions promote vaccination to reduce the risk for vaccine-preventable diseases (*1*). After 10 years of near 95% nationwide vaccination coverage, coverage with measles, mumps, and rubella vaccine (MMR)*; diphtheria, tetanus, and acellular pertussis vaccine (DTaP)^†^; poliovirus vaccine (polio)^§^; and varicella vaccine (VAR)^¶^ declined to approximately 93% over the 2020–21 and 2021–22 school years and remained essentially unchanged during the 2022–23 school year (*2*). These declines persisted after impacts of the COVID-19 pandemic diminished. This analysis summarizes state and local immunization program** data on vaccination coverage and exemptions from vaccination among kindergartners as reported to CDC by 49 states^††^ and the District of Columbia (DC), and provisional enrollment or grace period status for kindergartners reported by 31 states^§§^ for the 2023–24 school year.

## Methods

### Data Collection and Reporting

In compliance with state and local school entry requirements, parents provide children’s vaccination or exemption documents to schools, or schools obtain records from the state immunization information system (IIS). Federally funded immunization programs work with departments of education, local health departments, and school personnel to assess the vaccination and exemption status of children enrolled in public and private kindergartens. Programs report unweighted counts, aggregated by school type, to CDC via a questionnaire in the Secure Access Management System, a federal, web-based platform that provides authorized personnel access to public health applications operated by CDC. CDC uses these data to produce state- and national-level estimates of vaccination coverage among children in kindergarten. During the 2023–24 school year, 49 states and DC reported coverage with all state-required vaccinations and exemption data for public school kindergartners; 48 states and DC reported data for private school kindergartners.^¶¶^ Data from cities were included with their state data. State-level, national, and median coverage with the state-required number of DTaP, MMR, polio, and VAR doses are reported. Hepatitis B vaccination coverage is not included in this report and is available at SchoolVaxView (*2*). Thirty-one states reported the number of kindergartners attending school under a grace period (attendance without proof of complete vaccination or exemption during a set number of days) or provisional enrollment (attendance while completing a catch-up vaccination schedule). All counts were current at the time of the assessment by the immunization program.***

### Data Analyses

National estimates, medians, and summary measures include only 49 U.S. states and DC. Coverage and exemption estimates were adjusted based on survey type and response rate.^†††^ Results for U.S. territories and freely associated states are reported separately. National estimates measure coverage and exemptions among all kindergartners, whereas medians indicate the midpoint of state-level estimates. During the 2023–24 school year, immunization programs reported 3,823,472 children enrolled in kindergarten. Reported estimates are based on 3,559,990 (93.1%) children who were surveyed for vaccination coverage, 3,709,432 (97.0%) for exemptions, and 2,748,251 (71.9%) for grace period and provisional enrollment.^§§§^ Potentially achievable coverage with MMR (the sum of the percentage of children who were up to date with 2 MMR doses and those not up to date but nonexempt) was calculated for each jurisdiction. Students who were not up to date and did not have medical or nonmedical exemptions included those who were provisionally enrolled in kindergarten, in a grace period, or otherwise without documentation of complete vaccination. Required vaccines and required numbers of doses, methods and timing of data collection, and data reported varied by jurisdiction. Kindergartners were considered up to date with a given vaccine if they received all doses of that vaccine required for school entry, except in nine states^¶¶¶^ that reported kindergartners as up to date for any vaccine only if they had received all doses of all vaccines required for school entry. All but four states**** reported the number of kindergartners with an exemption from one or more vaccine. SAS software (version 9.4; SAS Institute) was used for all analyses. This activity was reviewed by CDC, deemed not research, and was conducted consistent with applicable federal law and CDC policy.^††††^

## Results

### Vaccination Coverage

Nationally, 2-dose MMR coverage was 92.7% (range = 79.6% [Idaho] to 98.3% [West Virginia]), with coverage ≥95% reported by 11 jurisdictions and <90% by 14 jurisdictions (Table). Five-dose DTaP coverage was 92.3% (range = 79.5% [Idaho] to 98.4% [West Virginia]); with coverage ≥95% reported by 12 jurisdictions and <90% by 15. Four-dose polio vaccination coverage was 92.6% (range = 80.1% [Idaho] to 98.4% [West Virginia]), with coverage ≥95% reported by 12 jurisdictions and <90% by 13. Two-dose VAR vaccination coverage was 92.4% (range = 79.1% [Idaho] to 99.7% [West Virginia]), with 10 jurisdictions reporting coverage ≥95% and 15 reporting <90% coverage. During the 2023–24 school year, coverage with each of the vaccines decreased in most states compared with that during the 2022–23 school year (Supplementary Figure 1, https://stacks.cdc.gov/view/cdc/164303).

**TABLE:** Estimated* coverage^†^ with measles, mumps, and rubella; diphtheria, tetanus, and acellular pertussis; poliovirus; and varicella vaccines; grace period or provisional enrollment^§^; and any exemption^¶^^,^** among kindergartners, by jurisdiction — United States,^††^ 2023–24 school year

Jurisdiction	Kindergarten population^§§^	Percentage							PP change in any exemption from last year to this year	Potentially achievable coverage****
		Surveyed^¶¶^	2 MMR doses***	5 DTaP doses^†††^	4 Polio doses^§§§^	2 VAR doses^¶¶¶^	Grace period or provisional enrollment	Any exemption		
National estimate^††††^	3,823,472	93.1	92.7	92.3	92.6	92.4	2.6	3.3	0.3	96.9
Median^††††^	—	—	92.0	91.3	91.7	91.8	2.0	3.7	0.4	96.4
**U.S. states and the District of Columbia**										
Alabama^§§§§,¶¶¶¶^	54,565	100.0	≥93.8	≥93.8	≥93.8	≥93.8	NP	2.2	0.2	97.8
Alaska^¶¶¶¶,^*****	8,644	88.9	84.3	83.8	84.4	82.6	NR	9.5	3.8	90.8
Arizona^†††††^	74,834	99.6	89.3	89.4	90.0	94.1	NR	8.5	1.1	92.8
Arkansas	37,535	95.4	92.5	91.4	91.4	92.0	8.0	3.5	0.4	96.6
California^§§§§,¶¶¶¶,†††††,§§§§§^	569,680	100.0	96.2	95.4	96.1	95.7	2.0	0.1	−0.1	99.9
Colorado^§§§§^	61,662	100.0	88.3	87.9	87.9	87.3	≥0.7	≥4.2	−0.1	96.0
Connecticut^§§§§,¶¶¶¶,†††††^	36,184	100.0	97.7	97.6	97.8	97.5	2.0	0.5	−0.3	99.5
Delaware^¶¶¶¶, §§§§§^	11,043	11.5	≥93.8	≥93.8	≥93.8	≥93.8	0.3	2.5	0.4	97.5
District of Columbia^§§§§,¶¶¶¶^	7,874	100.0	92.0	92.0	89.4	91.3	NR	2.3	1.0	97.9
Florida^§§§§,¶¶¶¶^	228,213	100.0	≥88.1	≥88.1	≥88.1	≥88.1	5.0	4.8	0.3	95.2
Georgia^§§§§,¶¶¶¶^	136,943	100.0	≥88.4	≥88.4	≥88.4	≥88.4	0.5	3.6	−0.2	96.4
Hawaii^¶¶¶¶^	13,995	8.2	89.8	89.4	90.2	88.9	5.6	5.3	−1.1	94.7
Idaho^§§§§^	22,376	100.0	79.6	79.5	80.1	79.1	1.4	14.3	2.2	86.5
Illinois^§§§§,¶¶¶¶^	133,578	100.0	91.6	91.2	91.5	91.1	NR	≥2.5	0.4	97.5
Indiana^¶¶¶¶,¶¶¶¶¶^	80,639	88.5	90.8	81.8	81.8	90.3	NR	2.4	−0.4	97.6
Iowa^§§§§,¶¶¶¶^	38,611	100.0	≥89.1	≥89.1	≥89.1	≥89.1	5.9	3.4	0.4	96.6
Kansas^¶¶¶¶,§§§§§,¶¶¶¶¶,^******	34,178	37.1	90.4	90.1	91.6	89.5	NP	3.0	0.1	97.0
Kentucky^§§§§,¶¶¶¶,§§§§§,¶¶¶¶¶^	52,609	100.0	≥90.0	≥91.0	≥91.7	≥89.4	NR	2.3	0.6	97.7
Louisiana^§§§§^	51,839	100.0	92.4	90.5	95.0	90.9	NP	2.8	0.5	97.2
Maine	12,087	93.0	97.5	96.9	97.4	97.1	1.1	1.0	0.1	99.4
Maryland^§§§§,¶¶¶¶,§§§§§^	63,224	100.0	≥96.6	≥96.8	≥97.0	≥96.2	NR	2.2	0.3	97.8
Massachusetts^§§§§,¶¶¶¶,§§§§§^	65,424	100.0	96.3	96.0	96.2	95.7	NP	1.4	0.0	98.7
Michigan^§§§§^	110,156	100.0	92.1	92.1	92.7	91.7	1.0	5.6	0.2	93.6
Minnesota	66,032	99.1	87.0	87.4	88.1	87.2	NR	≥5.4	0.9	94.6
Mississippi^§§§§,¶¶¶¶^	36,105	100.0	≥97.5	≥97.5	≥97.5	≥97.5	1.4	0.7	0.5	99.3
Missouri^§§§§,¶¶¶¶^	69,014	100.0	90.4	90.5	91.0	89.9	NR	≥4.7	0.9	95.3
Montana	NR	NR	NR	NR	NR	NR	NR	NR	NA	NA
Nebraska^§§§§,¶¶¶¶,§§§§§^	23,118	100.0	93.9	95.2	95.7	91.2	2.6	3.5	0.9	96.7
Nevada^¶¶¶¶^	31,261	91.3	91.9	91.1	91.5	91.2	2.9	6.7	1.1	94.0
New Hampshire^§§§§,¶¶¶¶,¶¶¶¶¶^	11,871	100.0	≥89.2	≥89.2	≥89.2	≥89.2	4.3	4.1	0.7	95.9
New Jersey^§§§§,¶¶¶¶,¶¶¶¶¶^	105,408	100.0	≥93.2	≥93.2	≥93.2	≥93.2	1.0	3.9	0.7	96.1
New Mexico^§§§§,¶¶¶¶^	20,699	100.0	95.0	94.8	95.0	94.4	2.0	1.6	0.1	98.4
New York (including NYC)^¶¶¶¶,†††††^	200,894	98.8	97.7	96.8	97.1	97.1	2.2	0.1	0.0	99.9
NYC^¶¶¶¶,†††††^	85,360	99.3	96.7	95.3	95.7	95.9	2.4	0.1	0.0	100.0
North Carolina^¶¶¶¶,§§§§§,¶¶¶¶¶^	125,964	90.8	93.8	93.5	93.8	93.3	1.4	2.9	0.5	97.3
North Dakota	9,674	97.8	91.0	90.6	91.1	90.8	NR	6.4	1.3	93.6
Ohio	133,716	94.0	89.2	89.3	89.6	88.5	6.5	4.2	0.4	96.2
Oklahoma^§§§§§^	49,979	93.8	88.3	89.0	91.4	93.7	NR	5.7	1.0	94.6
Oregon^§§§§, §§§§§^	39,568	100.0	91.2	90.5	91.1	93.9	NR	8.9	0.7	92.4
Pennsylvania	137,593	96.5	93.5	93.8	93.4	93.2	2.0	4.2	0.4	95.8
Rhode Island^¶¶¶¶,§§§§§,¶¶¶¶¶^	10,539	97.8	97.1	96.9	96.7	96.6	0.7	1.7	0.2	98.5
South Carolina ^¶¶¶¶,^******	58,069	26.7	92.1	92.3	92.6	91.8	4.5	4.4	0.3	95.6
South Dakota^¶¶¶¶,¶¶¶¶¶^	11,744	99.8	90.8	90.6	91.0	90.3	NR	5.7	1.6	94.3
Tennessee^¶¶¶¶,¶¶¶¶¶^	79,323	95.8	95.1	94.6	94.6	94.6	2.0	3.6	0.4	96.4
Texas (including Houston)^§§§§§,¶¶¶¶¶^	381,421	92.4	94.3	94.0	94.2	93.7	1.8	3.9	0.4	96.4
Houston^§§§§§,¶¶¶¶¶^	37,882	67.7	93.6	93.4	93.6	92.8	0.8	2.7	0.4	97.6
Utah^§§§§^	46,228	100.0	88.8	88.5	88.2	88.2	4.2	9.3	1.2	91.4
Vermont^§§§§,¶¶¶¶^	5,630	100.0	92.9	92.7	92.6	92.5	5.6	4.0	0.4	92.9
Virginia^¶¶¶¶,^******	92,633	1.9	94.2	96.2	93.0	93.1	NR	2.4	0.2	97.6
Washington^¶¶¶¶¶^	84,053	97.2	91.3	90.2	90.4	90.0	1.3	4.8	0.8	96.0
West Virginia^¶¶¶¶,†††††,§§§§§,¶¶¶¶¶^	18,261	82.8	98.3	98.4	98.4	99.7	NR	<0.1	0.0	99.9
Wisconsin^§§§§§,¶¶¶¶¶^	62,028	98.2	84.8	85.7	86.3	84.3	6.4	8.0	0.8	93.2
Wyoming^§§§§,¶¶¶¶^	6,754	100.0	93.5	92.2	92.5	95.1	2.0	5.6	0.8	94.6
**Territories and freely associated states**										
American Samoa^§§§§,¶¶¶¶,†††††^	758	100.0	78.0	76.8	75.3	76.4	NR	0	NA	100.0
Federated States of Micronesia^§§§§,¶¶¶¶,†††††,§§§§§^	1,578	100.0	91.3	77.7	79.7	Nreq	NR	0	NA	100.0
Guam^¶¶¶¶,¶¶¶¶¶^	1,936	97.8	93.0	89.7	92.1	Nreq	NR	0.2	NA	99.8
Marshall Islands^§§§§,¶¶¶¶,†††††^	947	100.0	99.5	93.6	92.9	Nreq	NR	NR	NA	NA
Northern Mariana Islands^§§§§,¶¶¶¶^	665	100.0	97.3	94.7	95.8	95.5	NR	0.2	0.2	99.8
Palau^§§§§,¶¶¶¶,§§§§§^	187	100.0	100.0	100.0	100.0	Nreq	NR	0	0	100.0
Puerto Rico^¶¶¶¶^	20,967	8.8	94.3	98.4	9842	94.3	NR	3.0	1.9	96.9
U.S. Virgin Islands^¶¶¶¶,§§§§§^	977	97.7	92.1	71.8	77.3	82.6	NR	4.8	NA	99.9

**Abbreviations:** DTP = diphtheria and tetanus toxoids and pertussis vaccine; DTaP = diphtheria, tetanus, and acellular pertussis vaccine; MMR = measles, mumps, and rubella vaccine; NA = not available; NP = no grace period or provisional policy; NR = not reported to CDC; Nreq = not required; NYC = New York City; polio = poliovirus vaccine; PP = percentage point; VAR = varicella vaccine.

* Estimates adjusted for nonresponse and weighted for sampling where appropriate.

^†^ Estimates based on a completed vaccination series (i.e., not vaccine specific) use the “≥” symbol. In Maryland, undervaccinated children might have been counted more than once by some schools; therefore coverage estimates use the “≥” symbol. Coverage might include history of disease or laboratory evidence of immunity. In Kentucky, public schools reported numbers of children up to date with specific vaccines, and most private schools reported numbers of children who received all doses of all vaccines required for school entry.

^§^ A grace period is a set number of days during which a student can be enrolled and attend school without proof of complete vaccination or exemption. Provisional enrollment allows a student without complete vaccination or exemption to attend school while completing a catch-up vaccination schedule. In states with one or both of these policies, the estimates represent the number of kindergartners who were within a grace period, were provisionally enrolled, or were in a combination of these categories.

^¶^ Some jurisdictions did not report the number of children with exemptions but instead reported the number of exemptions for each vaccine, which could count some children more than once. Lower bounds of the percentage of children with any exemptions were estimated using the individual vaccines with the highest number of exemptions. Estimates based on vaccine-specific exemptions use the “≥” symbol.

** Exemptions, grace period or provisional enrollment, and vaccine coverage status might not be mutually exclusive. Some children enrolled under a grace period or provisional enrollment might be exempt from one or more vaccinations, and children with exempt from one or more vaccines, and children with exemptions might be fully vaccinated with one or more required vaccine.

^††^ Includes five territories and three freely associated states.

^§§^ The kindergarten population is an approximation provided by each program.

^¶¶^ The number surveyed represents the number surveyed for coverage. Exemption estimates are based on 28,678 kindergartners for Kansas, 58,069 for South Carolina, and 92,606 for Virginia.

*** Most states require 2 doses of MMR; Alaska, New Jersey, and Oregon require 2 doses of measles, 1 dose of mumps, and 1 dose of rubella vaccines. Georgia, New York, NYC, North Carolina, and Virginia require 2 doses of measles and mumps vaccines and 1 dose of rubella vaccine. Iowa requires 2 doses of measles vaccine and 2 doses of rubella vaccine. Wyoming requires 1 dose of MMR.

^†††^ Pertussis vaccination coverage might include some DTP doses if administered in another country. Most states require 5 doses of DTaP for school entry, or 4 doses if the fourth dose was received on or after age 4 years; Maryland and Wisconsin require 4 doses; Nebraska requires 3 doses. The reported coverage estimates represent the percentage of kindergartners with the state-required number of DTaP doses, except for Kentucky, which requires ≥5 but reports ≥4 doses of DTaP. Wyoming requires 4 doses of DTaP.

^§§§^ Most states require 4 doses of polio for school entry, or 3 doses if the fourth dose was received on or after age 4 years; Maryland, Nebraska, and Wyoming require 3 doses. The reported coverage estimates represent the percentage of kindergartners with the state-required number of polio doses, except for Kentucky, which requires ≥4 but reports ≥3 doses of polio.

^¶¶¶^ Most states require 2 doses of VAR for school entry; Alabama, Arizona, New Jersey, Oklahoma, Oregon, and Wyoming require 1 dose. Reporting of VAR status for kindergartners with a history of varicella disease varied within and among states; some kindergartners were reported as vaccinated against varicella and others as medically exempt.

**** Potentially achievable coverage is estimated as the sum of the percentage of students with up-to-date MMR and the percentage of students without up-to-date MMR and without a documented vaccine exemption. The exemptions used to calculate the potential increase in MMR coverage for Alaska, Arizona, Arkansas, Colorado, District of Columbia, Idaho, Illinois, Maine, Massachusetts, Michigan, Minnesota, Missouri, Nebraska, Nevada, New York, North Carolina, Oklahoma, Oregon, Rhode Island, Texas, Utah, Vermont, Washington, Wisconsin, and Wyoming are the number of children with exemptions specifically for MMR. For all other jurisdictions, numbers are based on an exemption from any vaccine.

^††††^ National coverage, exemption estimates, and medians were calculated using data from 49 states and the District of Columbia (Montana, American Samoa, Guam, Marshall Islands, Federated States of Micronesia, Northern Mariana Islands, Palau, Puerto Rico, and the U.S. Virgin Islands were not included). Data from cities were included with their state data. National grace period or provisional enrollment estimates and median were calculated using data from the 31 states that have either a grace period or provisional enrollment policy and reported relevant data to CDC. Data reported from 3,559,990 kindergartners were assessed for coverage, 3,709,432 for exemptions, and 2,748,251 for grace period or provisional enrollment. Estimates represent rates for populations of coverage and exemptions (3,823,472) and grace period or provisional enrollment (2,839,159).

^§§§§^ The proportion surveyed is reported as 100% but might be <100% if based on incomplete information about the actual current enrollment.

^¶¶¶¶^ Philosophical exemptions were not allowed.

***** Reported public school data only.

^†††††^ Religious exemptions were not allowed.

^§§§§§^ Counted some or all vaccine doses received regardless of Advisory Committee on Immunization Practices recommended age and time interval; vaccination coverage rates reported might be higher than those for valid doses.

^¶¶¶¶¶^ Did not include certain types of schools, such as kindergartens in child care facilities, online schools, correctional facilities, or those located on military bases or tribal lands.

****** Vaccination coverage data were collected from a sample of kindergartners; exemption data were collected from a census of kindergartners.

### Vaccination Exemptions, Grace Period, and Provisional Enrollment

**FIGURE 1:**
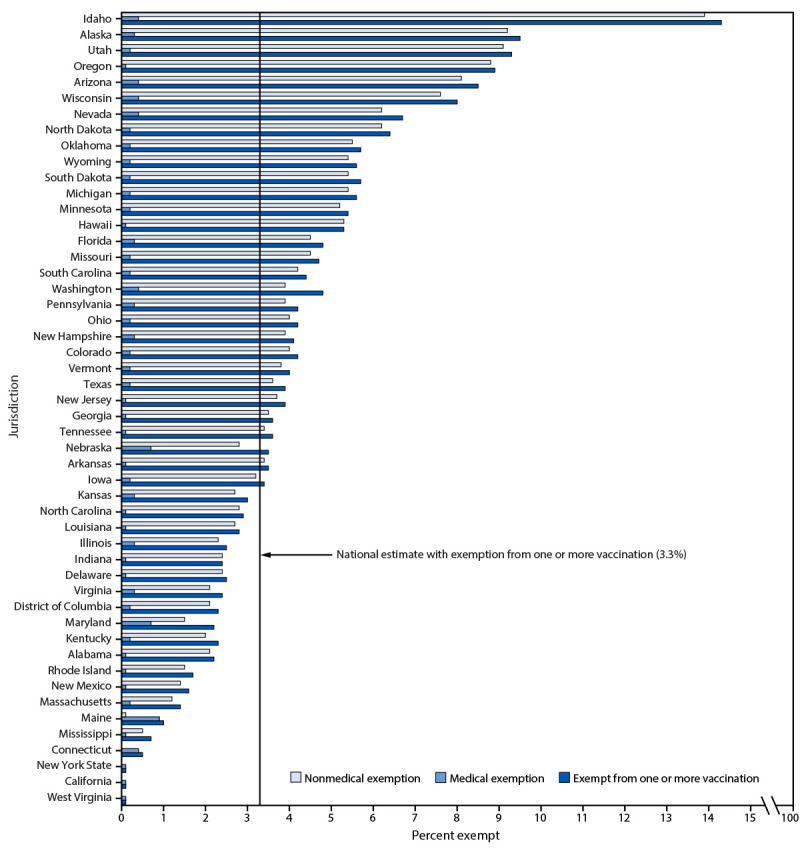
Estimated percentage*^,^^†^ of kindergartners with medical or nonmedical exemptions from one or more vaccination, by jurisdiction^§^ — United States, 2023–24 school year * Colorado, Illinois, Minnesota, and Missouri did not report the number of kindergartners with an exemption but instead reported the number of exemptions for each vaccine, which could have counted some children more than once. For these states, the percentage of kindergartners exempt from the vaccine with the highest number of exemptions by exemption type (the lower bound of the potential range of exemptions) was included in the national and median exemption rates. ^†^ Washington was unable to deduplicate data for students with both religious and philosophical exemptions; therefore, the nonmedical exemption type with the highest number of kindergartners (the lower bound of the potential range of nonmedical exemptions) was included in the national and median exemption rates for nonmedical exemptions. The percentage of kindergartners exempt from one or more vaccination is greater than the sum of the number of students with a medical exemption and the lower bound estimate of the number with a nonmedical exemption. ^§^ Montana did not report data for the 2023–24 school year.

**FIGURE 2:**
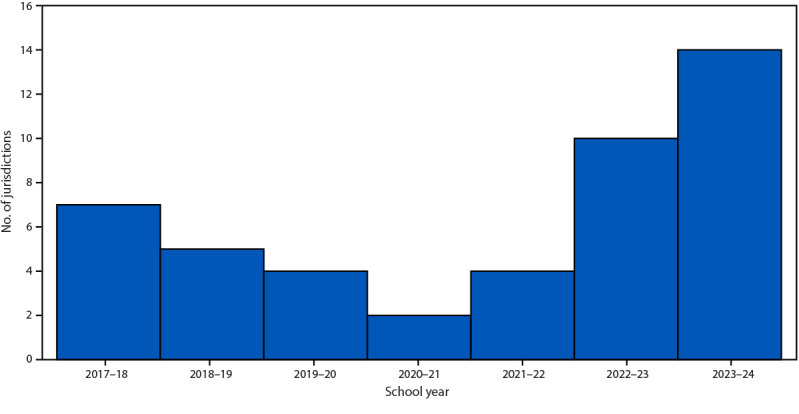
Number of jurisdictions that could not potentially achieve ≥95% coverage*^,^^†^ with measles, mumps, and rubella vaccine among kindergartners — United States, 2017–18 to 2023–24 school years **Abbreviations**: MMR = measles, mumps, and rubella vaccine; UTD = up to date. * Potentially achievable coverage is estimated as the sum of the percentage of students with UTD MMR and the percentage of students without UTD MMR and without a documented vaccine exemption. Montana did not report kindergarten vaccination coverage for 2021–22 through 2023–24 school years and is excluded from this analysis. ^†^ The exemptions used to calculate the potential increase in MMR coverage for Alaska, Arizona, Arkansas, Colorado, District of Columbia, Idaho, Illinois, Maine, Massachusetts, Michigan, Minnesota, Missouri, Nebraska, Nevada, New York, North Carolina, Ohio, Oklahoma, Oregon, Rhode Island, Texas, Utah, Vermont, Washington, West Virginia, Wisconsin, and Wyoming are the number of children with exemptions specifically for MMR. For all other states, numbers are based on an exemption for any vaccine.

Nationwide, 3.3% of kindergartners had an exemption (0.2% medical and 3.1% nonmedical^§§§§^) from ≥1 required vaccine (i.e., not limited to MMR, DTaP, polio, and VAR) in 2023–24 (range = <0.1% [West Virginia] to 14.3% [Idaho]) (Supplementary Table, https://stacks.cdc.gov/view/cdc/164305), compared with 3.0% during the 2022–23 school year (*2*). Nonmedical exemptions account for >93% of reported exemptions, and for almost 100% of the increase in the national exemptions (*2*). Exemptions from ≥1 vaccine were higher than the national exemption estimate of 3.3% in 30 states, and exemptions exceeded 5% in 14 states (Figure 1). States with increases in exemptions were distributed across all U.S. Department of Health and Human Services regions (Supplementary Figure 2, https://stacks.cdc.gov/view/cdc/164304). Nationwide, 4.0% of kindergarten students were neither fully vaccinated with MMR nor exempt, and the potentially achievable coverage nationally was 96.9%. Compared with previous years, fewer jurisdictions can potentially achieve 95% MMR coverage because of increasing exemptions: during 2020–21, two jurisdictions could not potentially achieve ≥95% MMR coverage compared with 14 jurisdictions during 2023–24 (Figure 2). Provisional kindergarten enrollment or grace period attendance was 2.6% among 31 states reporting these data (range = 0.3% [Delaware] to 8.0% [Arkansas]) (Table).

## Discussion

Nationwide, vaccination coverage among children in kindergarten decreased to <93% during the 2023–24 school year, remaining below the Healthy People 2030 MMR target of 95% (*3*) for the fourth consecutive year. As recently as the 2019–20 school year, coverage with each measured vaccine was 95% (*2*). Coverage with all four vaccines declined in 80% of jurisdictions. CDC’s Let’s RISE initiative (https://www.cdc.gov/vaccines/partners/routine-immunizations-lets-rise.html) supports jurisdictions with the largest declines in coverage with communication and programmatic efforts to improve coverage.

The percentage of U.S. kindergartners with an exemption from ≥1 vaccine increased to 3.3% (the highest percentage ever reported) (*2*), increased in 41 jurisdictions, and exceeded 5% in 14. The decreases in coverage, combined with increases in exemptions, jeopardize reaching the Healthy People 2030 95% coverage of kindergartners with MMR target. The number of jurisdictions with exemption rates >5%, making them unable to achieve ≥95% MMR coverage even if every nonexempt kindergartner was vaccinated, increased from two in 2020–21 to 14 in 2023–24. Approximately 280,000 (7.3%) kindergartners did not have documentation of 2 MMR doses and were potentially at risk for measles infection.

### Limitations

The findings in this report are subject to at least four limitations. First, conditions that include the vaccines and number of doses required, assessment date, acceptable documentation, data collection methods, allowable types of exemptions, and grace period and provisional enrollment policies vary, limiting comparisons among jurisdictions. Second, representativeness might be negatively affected by data collection methods that assess vaccination status at different times, or miss some schools or students (e.g., homeschooled students). Third, inaccurate, incomplete, or missing documentation could result in under- or overestimation of coverage, exemption rates, grace periods, or provisional enrollment. Finally, national coverage estimates for the 2023–24 school year include only 49 of 50 states and DC, 10 of which report lower bound estimates; exemption estimates include 49 states and DC, four of which report lower bound estimates.

### Implications for Public Health Practice

Among kindergarten students, vaccination coverage continues to decline as exemptions increase, setting the stage for accumulation of clusters of undervaccinated children, which can lead to outbreaks (*4*–*6*). These shifts underscore the importance of immunization programs, schools, and providers in ensuring that children are fully vaccinated before school entry. The Vaccines for Children program (https://www.cdc.gov/vaccines-for-children/about/index.html) helps to maintain vaccination coverage among children who are uninsured, have public insurance, or are American Indian or Alaska Native. In previous years, nearly all states had the potential to achieve ≥95% coverage if all nonexempt students were vaccinated, but increases in the percentage of students with exemptions have reduced that number to 36 (72%) in 2023–24. In a 2024 survey of U.S. parents, 8.3% disagreed with the statement that school and child care “vaccination requirements for children are important and necessary,” similar to the percentage of children not fully vaccinated; another 15.2% of parents had no opinion (*7*). These results could indicate changes in attitudes toward routine vaccination transferring from hesitancy about COVID-19 vaccination, or toward any vaccine requirements arising from objections to COVID-19 vaccine mandates, as well as a potential for larger decreases in coverage or increases in exemptions.

States have applied various approaches to increase vaccination or decrease exemptions, including reducing the types of exemptions available (e.g., medical only), requiring that exemption forms be notarized (*8*), and assuring consistency of school practices with state laws (*9*). Approaches known to increase vaccination coverage include enforcement of school vaccination requirements, school-based vaccination clinics, reminder and recall systems, strong provider recommendations, and follow-up of undervaccinated students (*10*). Schools can also work with parents to avoid exemptions because of difficulty meeting vaccination requirements deadlines and share vaccination and exemption data as part of school vaccination assessments by state and local health departments, in accordance with state requirements and as allowed by federal law, to monitor annual vaccination and exemption rates. While following evidence-based practices to improve vaccination coverage, providers could educate parents specifically about the safety and effectiveness of vaccinations required for school entry, and the risks of delayed or incomplete vaccination or nonvaccination to children, family members, classmates, and the community.

## Footnotes

* Jurisdictions reported coverage with all doses of MMR required by the jurisdiction. All states except Wyoming require 2 doses of a measles-containing vaccine. Seven states (Alaska, Georgia, New Jersey, New York, North Carolina, Oregon, and Virginia) require only 1 dose of rubella vaccine. Alaska, New Jersey, and Oregon require only 1 dose of mumps vaccine; mumps vaccine is not required in Iowa. Wyoming requires 1 dose of MMR for kindergarten entry, allowing students until the day before their seventh birthday to receive their second dose.

† Jurisdictions reported coverage with all doses of DTaP required by the jurisdiction. Nebraska requires 3 doses of DTaP; Maryland and Wisconsin require 4 doses; Wyoming requires 4 doses of DTaP for kindergarten entry, allowing students until the day before their seventh birthday to receive their fifth dose; all other states require 5 doses, unless dose 4 was administered on or after the fourth birthday. The reported coverage estimates represent the percentage of kindergartners with the state-required number of DTaP doses, except for Kentucky, which requires 5 doses of DTaP by age 5 years but reported 4-dose coverage for kindergartners.

§ Jurisdictions reported coverage with all doses of polio required by the jurisdiction. Two states (Maryland and Nebraska) require only 3 doses of polio; Wyoming requires 3 doses of polio for kindergarten entry, allowing students until the day before their seventh birthday to receive their fourth dose; all other states require 4 doses unless the last dose was given on or after the fourth birthday. The reported coverage estimates represent the percentage of kindergartners with the state-required number of polio doses, except for Kentucky, which requires ≥4 doses but reported ≥3 doses of polio.

¶ Jurisdictions reported coverage with all doses of VAR required by the jurisdiction. Five states require 1 dose of VAR; 44 states and DC require 2 doses. Wyoming requires 1 dose of VAR for kindergarten entry, allowing students until the day before their seventh birthday to receive their second dose.

** Federally funded immunization programs are in 50 states and DC, five cities, and eight U.S. territories and freely associated states. Two cities (Houston, and New York City) reported data to CDC, which were also included in data submitted by their state. State-level data were used to calculate national estimates and medians. Immunization programs in U.S. territories reported vaccination coverage and exemptions, reported in the Table and Supplementary Table; however, these data were not included in national estimates calculations.

†† Montana did not report school vaccination data.

§§ Arkansas, California, Colorado, Connecticut, Delaware, Florida, Georgia, Hawaii, Idaho, Iowa, Maine, Michigan, Mississippi, Nebraska, Nevada, New Hampshire, New Jersey, New Mexico, New York, North Carolina, Ohio, Pennsylvania, Rhode Island, South Carolina, Tennessee, Texas, Utah, Vermont, Washington, Wisconsin, and Wyoming reported data on the number of students within a grace period or provisionally enrolled at the time of assessment.

¶¶ Thirteen states reported coverage and exemption data for at least some homeschooled kindergartners, either separately, or included with data from public or private schools.

*** Assessment date varied by state and area. Three states assessed schools on the first day of school; eight states assessed schools by December 31; 17 states and DC assessed schools by some other date, ranging from October 1, 2023, to June 2, 2024; and 21 states assessed schools on a rolling basis.

††† Immunization programs that used census or voluntary response provided CDC with data aggregated at the state or local (city or territory) level. Estimates based on these data were adjusted for nonresponse using the inverse of the response rate, stratified by school type (public, private, and homeschool, where available). Programs that used complex sample surveys provided CDC with data aggregated at the school or county level for weighted analyses. Weights were calculated to account for sample design and adjusted for nonresponse.

§§§ These totals are the sums of the kindergartners surveyed among programs reporting data for coverage, exemptions, grace periods, and provisional enrollment.

¶¶¶ Alabama, Delaware, Florida, Georgia, Iowa, Mississippi, New Hampshire, and New Jersey considered kindergartners up to date only if they had received all doses of all vaccines required for school entry. In Kentucky, public schools reported numbers of children up to date with specific vaccines, and most private schools reported numbers of children who received all doses of all vaccines required for school entry.

**** Colorado, Illinois, Minnesota, and Missouri did not report the number of kindergartners with an exemption but instead reported the number of exemptions for each vaccine, which could have counted some children more than once. For these states, the percentage of kindergartners exempt from the vaccine with the highest number of exemptions by exemption type (the lower bound of the potential range of exemptions) was included in the national and median exemption rates.

†††† 45 C.F.R. part 46.102(l)(2), 21 C.F.R. part 56; 42 U.S.C. Sect. 241(d); 5 U.S.C. Sect. 552a; 44 U.S.C. Sect. 3501 et seq.

§§§§ Washington was unable to deduplicate data for students with both religious and philosophical exemptions; therefore, the nonmedical exemption type with the highest number of kindergartners (the lower bound of the potential range of nonmedical exemptions) was included in the national and median exemption rates for nonmedical exemptions.
