# *Erratum*: Vol. 73, No. 41

**DOI:** 10.15585/mmwr.mm7425a3

**Published:** 2025-07-10

The report “Coverage with Selected Vaccines and Exemption Rates Among Children in Kindergarten — United States, 2023–24 School Year” contained several errors. 

On page 926 in the Data Analyses section, the following sentences should have read, “During the 2023–24 school year, immunization programs reported **3,823,506** children enrolled in kindergarten. Reported estimates are based on **3,560,047** (93.1%) children who were surveyed for vaccination coverage, **3,709,489** (97.0%) for exemptions, and **2,748,308** (71.9%) for grace period and provisional enrollment.^§§§^”

On page 929 in the Vaccine Exemptions, Grace Period, and Provisional Enrollment section, the following sentences should have read, “Nationwide, 4.0% of kindergarten students were neither fully vaccinated with MMR nor exempt, and the potentially achievable coverage nationally was **96.8%**. Compared with previous years, fewer jurisdictions can potentially achieve 95% MMR coverage because of increasing exemptions: during 2020–21, two jurisdictions could not potentially achieve ≥95% MMR coverage compared with **13** jurisdictions during 2023–24 (Figure 2).”

On page 929 in the Vaccine Exemptions, Grace Period, and Provisional Enrollment section, the following sentences should have read, “The number of jurisdictions with >5% **of kindergartners exempt from any vaccine increased from two in 2020–21 to 14 in 2023–24. The number with >5% of kindergartners exempt specifically from MMR,** making **those jurisdictions** unable to achieve ≥95% MMR coverage even if every nonexempt kindergartner were vaccinated, increased from two to **13**.”

On page 927, the Table contained multiple errors. In the row “National estimate” the value under the column “Kindergarten population” should have been **3,823,506** and, in that same row, the value under the column “Potentially achievable coverage” should have been **96.8**. In the row “Delaware” the value under the column “Surveyed” should have been **12.1**. In the row “Hawaii” the value under the column “2 VAR doses” should have been **89.1**. In the row “North Dakota” the values under the columns “Kindergarten population,” “Surveyed,” “2 MMR doses,” “5 DTaP doses,” “4 Polio doses,” “2 VAR doses,” “Any exemption,” “PP change in any exemption from last year to this year,” and “Potentially achievable coverage” should have been **9,708**, **97.5**, **90.9**, **90.5**, **91.0**, **90.7**, **6.5**, **1.4**, and **93.5,** respectively. In the row “Pennsylvania” the values under the columns “Any exemption,” “PP change in any exemption from last year to this year,” and “Potentially achievable coverage” should have been **5.0**, **0.9**, and **95.0**, respectively. In the row “Vermont” the value under the column “Potentially achievable coverage” should have been **96.0**. In the row “Puerto Rico” the value under the columns “4 Polio doses,” “Any exemption,” “PP change in any exemption from last year to this year,” and “Potentially achievable coverage” should have been **98.4**, **2.9**, **1.8**, and **97.1**, respectively. 

On page 928 in the Table, the last sentence in the 13th footnote should have read, “The exemptions used to calculate the potential increase in MMR coverage for Alaska, Arizona, Arkansas, Colorado, District of Columbia, Idaho, Illinois, Maine, Massachusetts, Michigan, Minnesota, Missouri, Nebraska, Nevada, New York, North Carolina, Oklahoma, Oregon, Rhode Island, Texas, Utah**, **Washington, Wisconsin, and Wyoming are the number of children with exemptions specifically for MMR. For all other jurisdictions, numbers are based on an exemption from any vaccine.” The last two sentences of the 14th footnote should have read, “Data reported from **3,560,047** kindergartners were assessed for coverage, **3,709,489** for exemptions, and **2,748,308** for grace period or provisional enrollment. Estimates represent rates for populations of coverage and exemptions (**3,823,506**) and grace period or provisional enrollment (2,839,159).”

